# Reducing Motion Artifact in High Resolution 7 T MRI Using the Magnetic Resonance Minimal Motion (“MR‐MinMo”) Head Stabilization Device

**DOI:** 10.1002/mrm.70424

**Published:** 2026-06-02

**Authors:** Jyoti Mangal, Simon Richardson, Yannick Brackenier, Matthew Gardner, Pierluigi Di Cio, Chiara Casella, Shaihan Malik, Jo Hajnal, Martina F. Callaghan, Fred Dick, David W. Carmichael

**Affiliations:** ^1^ Research Department of Imaging Physics and Engineering, School of Biomedical Engineering and Imaging Sciences King's College London London UK; ^2^ Division of Psychology and Language Sciences University College London London UK; ^3^ Wellcome Centre for Human Neuroimaging University College London London UK

**Keywords:** head MRI, motion reduction, MRI hardware, ultra‐high field MRI

## Abstract

**Purpose:**

To evaluate the effectiveness of the MR‐MinMo head stabilization device in mitigating motion artifacts during long‐duration, high‐resolution 7 T MRI scans, with and without retrospective motion correction

**Methods:**

The MR‐MinMo was tested on seven pediatric and 12 adult healthy volunteers using 0.6 mm isotropic, 3D Multi‐Echo Gradient Echo (ME‐GRE) scans. A ∼10‐min ME‐GRE scan (linear sampling acceleration factor 2 × 2) and a ∼20‐min scan (DISORDER sampling acceleration factor 1.4 × 1.4) were obtained. Both scans were acquired with and without the MR‐MinMo in a 2 × 2 factorial study design. Qualitative and quantitative assessment of image quality of the first echo image was obtained via visual inspection and the normalized gradient squared (NGS) metric, respectively. A repeated measures ANOVA was used to determine individual and combined effects of the device and DISORDER images with retrospective motion correction on NGS values, with age as a binary factor (pediatric/adult). T2* maps were generated and the standard deviation of white matter R2*(=1/T2*) values assessed across conditions.

**Results:**

The MR‐MinMo significantly reduced motion artifacts both visually and in terms of NGS scores, particularly in pediatric volunteers. There was a significant interaction between MR‐MinMo and DISORDER motion correction, suggesting that MR‐MinMo improved retrospective motion correction. T2* maps demonstrated improved visual appearance and reduced WM variance with the MR‐MinMo.

**Conclusion:**

The MR‐MinMo can improve image quality via motion reduction in high‐resolution 7 T scans. By keeping motion within a correctable regime, the device can also improve the performance of retrospective motion correction methods.

## 1 Introduction

Among imaging modalities, MRI is particularly sensitive to subject motion compared to alternatives like ultrasound or CT. This heightened sensitivity arises from the longer acquisition times required for most MRI sequences to gather sufficient data for faithful image formation. Since acquisition times are far longer than timescales of physiological, volitional and accidental subject motion, they often affect MRI image quality [1, 2]. When motion occurs at a spatial scale comparable to the imaging resolution, it introduces artifacts including reduced signal‐to‐noise ratio (SNR), distortion, blurring, and ghosting. Even in compliant volunteers, degradation may be caused by periodic involuntary motion, e.g., cardiac, respiratory and blood flow, aperiodic motion including yawning, swallowing, sneezing or coughing, and conscious movement, e.g., due to discomfort [3]. In children and in patients with neurological disorders motion tends to be exacerbated [4]. A useful summary of the effect of motions on MR images is provided by Zaitsev et al. [1].

Ultra‐high field MR (≥ 7 T) offers the advantage of increased SNR, enabling the acquisition of high‐resolution images. However, their acquisition requires longer scan times, making them even more susceptible to motion‐induced degradation. Reduced voxel dimensions also increase sensitivity to finer scale subject motion. Strong interactions at ultra‐high fields may pose additional challenges—motion‐induced static magnetic (*B*_0_) field changes [5] increase due to greater susceptibility effects, while applied radio frequency (*B*_1_) fields also change related to movement [6] due to stronger coupling between the subject and RF field, increasing the complexity of motion correction strategies.

Quantitative mapping such as R2* mapping is sensitive at 7 T and enables tissue characterization of varying iron content [7, 8] with increased specificity [7]. This may be important in several neurological conditions including Parkinson's disease and epilepsy [8]. However, motion can severely degrade the precision and accuracy of the R2* estimates [9, 10, 11].

To fully realize the image quality benefits of 7 T MRI for clinical usage, it is crucial to develop practical solutions for reducing motion effects appropriate for pediatric and adult neuroimaging. To reduce motion at source by providing improved head stability and comfort, we designed and tested a new head stabilization device for high‐resolution 7 T neuroimaging, named *MR‐MinMo* [12]. The MR‐MinMo device used here is a research “in‐house” manufactured prototype yet to be made a commercial product. This work represents part of its performance validation process. The primary aim was to investigate if it improves retrospective motion correction. To test this, we used a self‐navigated sequence approach that does not require any additional hardware, namely Distributed and Incoherent Sample Orders for Reconstruction Deblurring using Encoding Redundancy (DISORDER) [13]. This sequence involves modified sampling of a Cartesian *k*‐space trajectory with an iterative retrospective reconstruction that includes a motion estimate. We hypothesized that the MR‐MinMo device would improve image quality for DISORDER retrospective motion correction by maintaining the data within a correctable regime.

## 2 Methods

### 2.1 The MR‐MinMo Device

The MR‐MinMo device used in this study is a head stabilization tool designed to reduce motion in awake participants, typically aged 6 and older. The device version used in this study was a prototype for comfortable head positioning inside the 8‐channel and single‐channel transmission 7 T RF head coils manufactured by Nova Medical (Wilmington, MA, USA).

**FIGURE 1:**
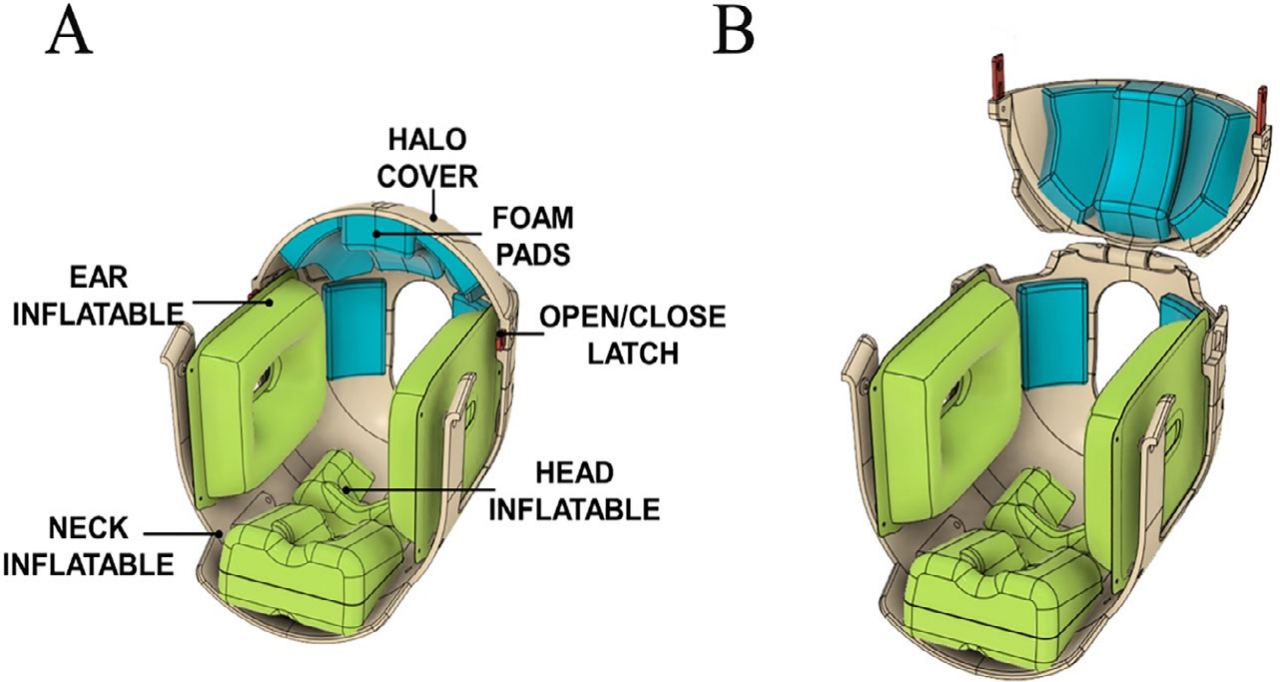
The schematic diagram of the MR‐MinMo with labeled components in (A) closed configuration and (B) open configuration. The device primarily consists of (i) Frame: the plastic structure that holds the other components in place within the head coil (ii) Halo: the upper unhinged section of the main Frame, (iii) Latch: the lock that holds the unhinged section of the main frame, (iv) Inflatables: the various plastic components that can be inflated or deflated around the ears, neck and head, (v) Padding system: consists of horn pad and mohawk pad made with memory foam, and (not shown) (vi) Manifold: the box that controls the air pressure distribution specific to each inflatable and (v) Vent: the valve mounted on the manifold which releases air from all inflatables at once when set to Open.

In brief, the MR‐MinMo consists of a 3D printed polycarbonate frame which conforms to the inner surface of the Nova coil and serves as mounting points for various functional modules. These modules are inflatables made of Polyurethane (PU) film and fabric‐covered pads made of PU foam with PU covers which are at set positions. The frame includes an articulated “halo” (also 3D printed polycarbonate) which hinges down, and latches closed to allow entry of the participant and rapid egress. The diagram of the device in its open and closed configurations is shown in Figure 1. The halo's latches can be unlocked and locked to change the device's configuration. When locked, the halo is latched to the lower frame in “closed configuration” and ready for scanning. When unlocked, the halo can be lifted for the device to be in “open configuration” and ready for loading/unloading the participant. Additionally, the use of a hairnet with the MR‐MinMo is recommended to distribute the gripping force on the head, to prevent hair being caught in the mechanism and to provide an improved feel compared to the plastic surface of the inflatables (images of the device with and without subject are provided in Figure S1). The MR‐MinMo has been designed with a quick‐release valve on the manifold to facilitate quick subject evacuation and also includes a relief valve to ensure pressure does not exceed safe levels. Most participants are able to have a clear line of sight out of the coil via a mirror. This enables standard patient distraction and facilitates functional imaging task‐based studies. The device was designed to ensure sufficient airflow and provide noncontacting areas to allow for temperature regulation.

### 2.2 Image Acquisition

To test the effectiveness of the MR‐MinMo device, we utilized a high‐resolution 3D ME‐GRE acquisition. Two types of *k*‐space sampling were used: (1) a standard (or conventional) linearly encoded acquisition and (2) a DISORDER encoded acquisition. The ME‐GRE acquisition was chosen because it facilitates T2* estimation, which in turn is sensitive to both bulkhead and physiological motion. The DISORDER trajectory served two purposes. First, it introduced a deliberately motion‐sensitive trajectory that is likely to produce artifacts even in compliant volunteers prior to motion correction, making it a more sensitive test of the MR‐MinMo device's capacity for reducing small‐scale motion. Second, it provided data that could be used for retrospective correction, thereby allowing for comparison of efficacy and potential synergistic application with the MR‐MinMo device.

The parameters used as a base for image acquisition paradigms were TR = 30 ms, flip angle = 36°, bandwidth = 470 Hz/px, number of echoes = 10, equally spaced at 2.68 ms intervals between TE1 = 2.27 ms and TE10 = 26.39 ms. Bipolar readouts were acquired with the elliptical shutter switched on for scan acceleration. A nonselective RF pulse was used, and echo readouts were optimized to minimize dead time. The scan had an isotropic resolution of 0.6 mm^3^, and a field of view (FOV) = AP/LR/HF = 256 × 173 × 218 mm^3^.

**FIGURE 2:**
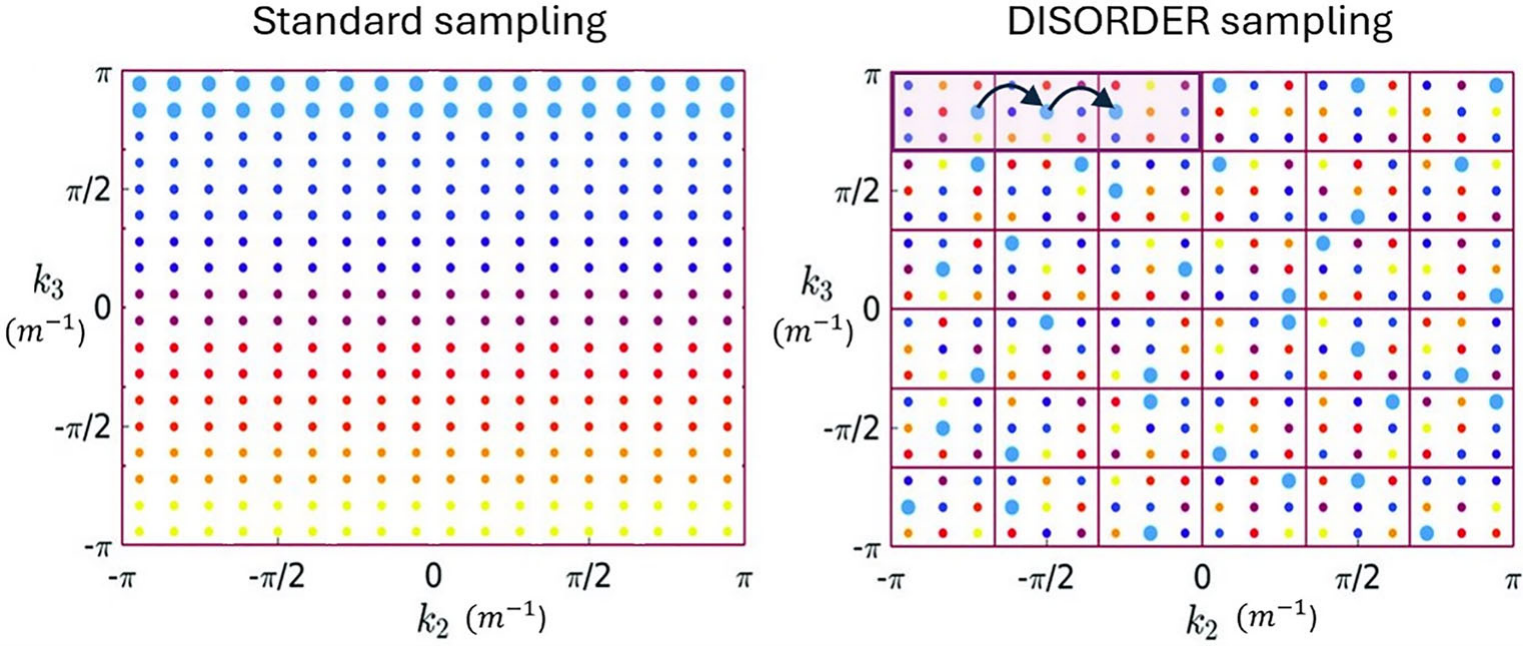
The different *k*‐space data acquisitions shown for visual representation. On the left, the standard sampling case in which adjacent lines in the phase encoding plane are acquired linearly. On the right, the DISORDER sampling case is shown for a tile size of 3 × 3 in which distributed information in *k*‐space is acquired with a pseudo‐random order of samples. Each color represents a different shot.

In the DISORDER encoding method (Figure 2), *k*‐space samples are acquired in a pseudo‐random order from rectangular regions or ‘tiles’ of the phase encoding plane of *k*‐space. Acquisition is performed in shots, where each shot consists of acquiring one sample from each tile within the phase‐encoding plane $k_{2}$ × $k_{3}$ and “shot duration” is defined as the time taken to acquire each shot. In Figure 2, each color represents a different shot. As shown in Cordero‐Grande et al. [13], each sample from within a tile is acquired pseudo‐randomly resulting in a distributed temporal coverage within each tile as well as for the whole *k*‐space spectrum. In this study, we employed the “random‐checkered” approach, with data acquired in the head‐foot (HF) $k_{1}$, anteroposterior (AP) $k_{2}$, and left–right (LR) $k_{3}$ orientations. Tiles of the sagittal plane are sampled faster within each shot, improving robustness to intra‐shot motion.

#### 2.2.1 DISORDER Scan Parameters

DISORDER relies on a degree of redundancy in acquired array coil data to reconstruct images and estimate motion states. Previous work by Cordero‐Grande et al. [13] empirically evaluated motion correction performance across different acceleration factors (table 1 in Cordero‐Grande et al. [13]) and validated the use of moderate acceleration factor 1.4 × 1.4 [14] (*R* = √2 × √2≈2) for robust motion correction by testing under large motion excursions (up to rotations of 25°). Further theory can be found in section 4.2 of Cordero‐Grande et al. [13]. This acceleration was replicated on the 7 T Siemens Terra scanner by over sampling in the phase encoding (PE) directions because only integer acceleration factors are allowed. To achieve the target acceleration of ∼1.4 × 1.4, a GRAPPA [15] acceleration of 2 was used in both PE directions, together with oversampling by 44% and 42% in each phase encoding direction, respectively. Oversampling percentages “P” were determined using the scanner‐permissible values, calculated according to the formula: $\frac{P}{100}+1=1.4$. The tile size for the DISORDER acquisition was set to 24 × 24 in the PE plane, implying a total of 576 points in each tile as well as 576 total shots or motion states in the *k*‐space, with a shot duration of 2.21 s. This tile size was selected within the scanner's allowable limits while ensuring quicker shots to optimize motion correction. Motion was estimated from *k*‐space data segments of six consecutive shots acquired from consecutive blocks. This was done to balance the timescale of motion states while remaining within practical computational times for 0.6 mm^3^ resolution images. The total acquisition time for the DISORDER encoded scan was 21:01 min, classified as a “very long scan.”

#### 2.2.2 Linear Sampling Scan Parameters

A high‐resolution scan was performed using the standard linear Cartesian sampling trajectory. This acquisition was designed to be faster and less sensitive to motion compared to the scan using DISORDER sampling. To achieve this, the acceleration factor was set to 2 × 2 with no oversampling in the phase encoding directions, providing an overall acceleration factor of 4. The total acquisition time for this scan was 10:38 min and while it was shorter than the DISORDER scan, it was classified as a “long scan.” This acquisition was chosen as typical for conventional ME‐GRE studies used for quantitative mapping [16].

To estimate coil sensitivity maps for offline reconstruction of the high‐resolution scans, a fully sampled reference GRE scan was acquired using the same field of view (FOV = 256 × 173 × 218 mm^3^) and adjustment volume. No oversampling was applied, and the elliptical shutter was switched on. The acquisition parameters were set to flip angle = 18°, TR = 5.3 ms, with a single echo readout at TE = 2.27 ms and bandwidth = 420 Hz/px. The resolution for the reference scan was 4.7 mm^3^, with a total acquisition time of 14 s. As for the high‐resolution scans, a nonselective RF pulse was used. The estimation of receiver coil sensitivity maps needed for the implementation of the reconstructions used the ESPIRiT algorithm. Coil sensitivities were estimated at low resolution and interpolated to the full 0.6 mm^3^ acquisition resolution using cubic spline interpolation (using MATLAB's “interpn” function). Phase singularities were addressed through magnitude‐weighted phase unwrapping and normalization by a virtual body coil reference calculated using the first principal component of the coil array data, ensuring that any potential phase discontinuities in low‐SNR regions did not affect the smooth coil sensitivity profiles.

### 2.3 Image Reconstruction

For all images, reconstruction was performed off‐line using code developed by the authors in MATLAB version R2019b (www.themathworks.com). All images were initially reconstructed using conjugate gradient SENSE (CG‐SENSE). The CG‐SENSE reconstruction was performed with a maximum of 300 iterations, with the tolerance level set to 0.001.

For DISORDER image reconstruction with motion correction, a least squares minimization problem known as the aligned‐SENSE [17] was solved as previously described [13]. In this approach, initialization is a CG‐SENSE reconstruction. Rigid body motion states, as well as the image data, are alternately minimized. Motion estimates are facilitated by the multiple views and images acquired from different coils. These views provide complementary information that helps constrain the motion estimation problem, enabling it to be solved iteratively.

For the motion estimation, the reconstruction framework used two resolution levels, defined as the pyramid plan by Cordero‐Grande et al. [13]. These levels corresponded to downscaled resolutions at factors [0.5, 1] of the total resolution resulting in voxel sizes of [1.2, 0.6 mm]. Motion parameters were estimated using 4 iterations at the first resolution level (1.2 mm voxel size). The final reconstruction was then performed at the higher resolution level (0.6 mm voxel size) using the motion parameters estimated from the first resolution level, with only 1 iteration performed at this resolution to maximize computational efficiency [13]. In addition to motion estimation, an outlier rejection step was incorporated to enhance the quality of the reconstruction. Outliers were identified based on the normalized median error for each motion state, enabling the pipeline to ignore data points likely affected by excessive motion. Motion estimation was performed using only the first echo (TE = 3.5 ms), as this provided the higher SNR, and the estimated motion states were then applied to correct all 10 echoes during reconstruction. The total reconstruction pipeline, in its current version, for the DISORDER dataset took approximately 8–10 h for level 1 and 20–30 h for level 2 depending on the motion correction pipeline. While the computation times can be long, they reflect the current algorithmic implementation at 0.6 mm^3^ with 576 motion states, with ongoing optimization work aiming to reduce processing time. Reconstructions were performed on a dual (8 × 2 cores) Intel(R) Xeon(R) @3.10Gz system with 256GB RAM.

All image volumes were corrected for bias field inhomogeneities using SPM12's bias correction algorithm [18, 19], processed via the “Segment” batch.

### 2.4 2 × 2 Factorial Study Design

**FIGURE 3:**
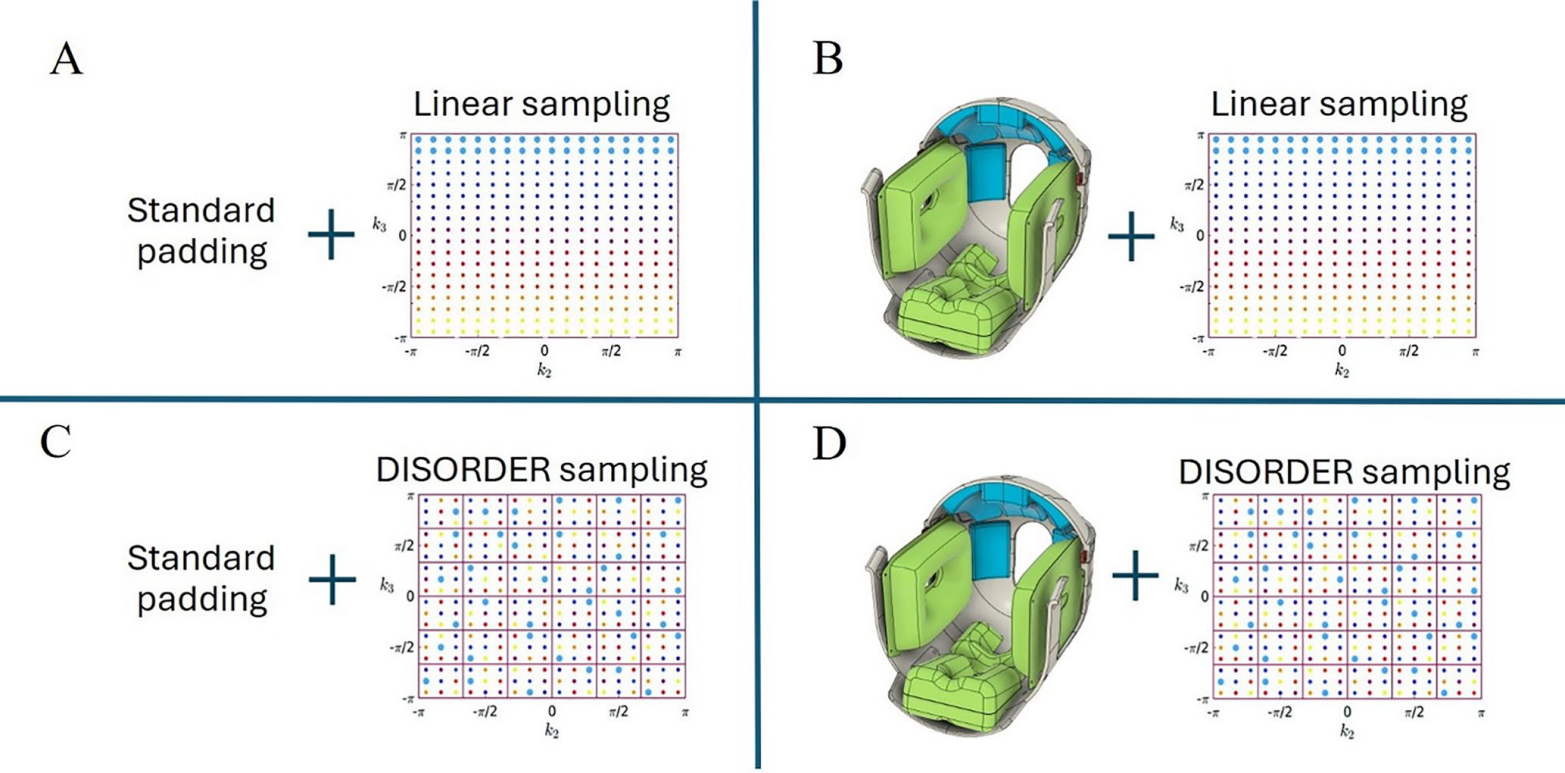
The 2 × 2 experimental scanning scheme. The independent variables of the scheme were MR‐MinMo and DISORDER with two levels of each, i.e., MR‐MinMo, standard padding and DISORDER, linear sampling, resulting in four conditions: (A) standard padding + linear sampling; (B) MR‐MinMo + linear sampling; (C) standard padding + DISORDER; and (D) MR‐MinMo + DISORDER. Standard padding implies the conventional padding set‐up was used. DISORDER referred here implies DISORDER trajectory and motion correction was used.

A 2 × 2 factorial design was used (see Figure 3) to assess the independent and interactive effects of using the MR‐MinMo device and DISORDER sampling with retrospective motion corrected reconstruction with the following four conditions: (A) Standard padding with linear sampling, (B) MR‐MinMo with linear sampling, (C) standard padding with DISORDER sampling and motion correction, and (D) MR‐MinMo with DISORDER sampling and motion correction. This allows the assessment of the effect of MR‐MinMo compared to standard padding and the potential additional benefit to reducing motion with MR‐MinMo at source prior to retrospective correction. Across all the healthy volunteers scanned, the order of using the MR‐MinMo and standard padding was randomized (MR‐MinMo first for 9/19 participants, second for 10/19 participants) to counterbalance any potential order effects, as well as the known interaction between duration of time in the scanner and participant motion [20]. While no independent motion tracking was used, the randomized order of the differently sampled scans ensures that any systematic changes in motion profiles with time should not have biased our comparisons between conditions.

### 2.5 MR‐MinMo Device Set‐Up

The MR‐MinMo device was used during scan acquisition in the 8‐channel transmit 7 T Nova coil in the closed configuration as shown in Figure 1A. While loading the participant with the device, constant feedback was obtained by the radiographer on participant comfort and stability. The hairnet size used by each participant was chosen with individual verbal feedback in the radiographer's presence to ensure the correctly sized hairnet was used.

Before loading the participant, the device was seated in the head coil in open configuration and was stabilized purely by a friction fit. The participant was asked to lie on the table while being guided to position their head in the device. Once the head was positioned into the device, the participant was instructed to shuffle the head down into the coil to avoid any empty spaces near the scanner‐end of the coil. Minimal air was pumped into the neck inflatable by squeezing the bulb twice lightly to give neck support while the participant was instructed to raise their chin slightly. The halo was then secured in the closed configuration, and the neck, ear and head inflatables were further inflated with air until the participant reported experiencing immobilization while still maintaining high comfort levels. While using the MR‐MinMo, no additional foam or cushioning was used.

The total time that the MR‐MinMo was used during a single scan session was ∼33 mins: a DISORDER acquisition (∼21 min), a standard acquisition (∼11 min), and a combination of reference acquisition and localizer (∼1 min). This was roughly half of the total scan session time, with the other half performed without the MR‐MinMo using the standard participant set‐up and padding used at our institution and the same image acquisition protocols. (Again, the order of MR‐MinMo and standard scan was pseudorandomized across participants.) Each scan session lasted ∼1 h and 15 min, with approximately 5–10 min to reposition the participant.

Participants were positioned by experienced research radiographers with and without the MR‐MinMo device. The standard padding (SP) involved the use of a manufacturer‐provided immobilization system (Siemens Healthineers) consisting of open‐celled polyurethane foam cushions configured for the 32‐channel recieve Nova head coils. This configuration included: a foam cushion positioned beneath the participant's head for support and comfort; viscoelastic memory foam pads positioned laterally on either side of the head within the coil housing, additional foam wedges placed above the head to fill remaining space between the participant and RF coil, and foam ear pads serving dual purpose for lateral support and some hearing protection in addition to foam earbuds. This standard padding configuration and approach represents both local and typical practice in our experience at 7 T imaging centers and therefore serves as an appropriate baseline.

For both setups, a cushion was placed under the knees for comfort and stabilization as conventionally used, and earbuds were provided for sound attenuation.

To minimize discomfort or boredom, all the pediatric participants watched TV (without sound) for the entire session via a projector external to the scan room and screen mounted within the scanner bore. Adult participants, many of whom were accustomed to MRI, were offered TV, although not all chose to watch.

### 2.6 Participants

Twenty participants were recruited for the study (12 adults and eight children). All participants were above the minimum weight requirement of 30 kg for the scanner. Out of the 20, 19 participants completed the scanning according to the 2 × 2 factorial design: 12 adults (ages 20–36 years, mean 28.25; seven females, five males) and seven children (ages 10–15 years, mean 12.14; two females, five males). The remaining one participant withdrew due to discomfort experinced during the standard set‐up condition which was performed first in this participant. Data from the linear Cartesian encoded acquisition was successfully processed for all 19 participants. For the DISORDER encoded acquisition, data from 16 participants (nine adults and seven children) were processed, with three adult datasets being unavailable due to technical raw data transfer issues. The pediatric participants were healthy volunteers (HVs) experiencing both 7 T MRI and the MR‐MinMo device for the first time. In contrast, most adult HVs were familiar with the research scanning environment and had previously undergone a 7 T MRI.

Informed consent was obtained from all participants or their legal representatives, as appropriate. All healthy adult scans were performed according to the local ethics approval (HR‐18‐19‐8700).

### 2.7 Image Quality Analyses

To assess the effects of both the MR‐MinMo device and DISORDER trajectory on image quality, analyses were performed on all reconstructed ME‐GRE image volumes (the shortest TE image, or echo 1, was used due to its highest SNR) and R2* maps. For the echo 1 image, the aim was to compare image quality across the different conditions in the 2 × 2 factorial design, using the normalized gradient squared (NGS) metric, which correlates closely with visual image quality assessment [21]. The NGS metric was calculated according to the formula following McGee et al. [21].

(1) $$\mathrm{NGS}=\sum_{\mathrm{ij}}{\left(\frac{\left|\;\left[\begin{array}{l} 1\\ -\;1 \end{array}\right]*g_{i,j}\;\right|}{\sum_{\mathrm{ij}}\left|\;\left[\begin{array}{l} 1\\ -\;1 \end{array}\right]*g_{i,j}\right|}\right)}^{2}$$

where $g_{i,j}$ is the pixel value at coordinate i,j in the image.

For image quality analysis, whole brain regions of interest (wbROIs) were extracted using SPM12. These were obtained using the scanner reconstructed first echo volume from the linear sampling and standard padding acquisition (condition A). This image was segmented using SPM12's unified segmentation algorithm with the default tissue probability maps in MNI space. The tissue classes (gray matter, white matter, and CSF) were determined to be within the brain if they had a probability greater than 0.9 of belonging to one of these tissue classes. While the tissue probability maps were derived from adult brains, the pediatric cohort, aged 10+, was within an age range where brain morphology (particularly white matter) is sufficiently similar to adult anatomy to derive a mask. To ensure a complete brain ROI, MATLAB function *imclose* was used to fill any gaps, and *imerode* with a disk element of radius 2 was applied to remove boundary pixels thus minimizing edge effects. The within‐wbROI mean NGS value was calculated for each of the four acquisitions resulting from the 2 × 2 factorial design.

### 2.8 Motion Parameters Analysis

To complement image quality assessment, motion states were extracted from the DISORDER motion correction reconstruction framework for all participants. DISORDER estimates rigid‐body motion (three translation and three rotations) for each motion state. To characterize intrascan motion, the temporal standard deviation was calculated across all 96 motion states for the six motion parameters. Average translation was computed as the average of the standard deviations of the three translational parameters (mm), and average rotation as the average of the standard deviations of the three rotational parameters (degrees/°). These metrics were determined for DISORDER scans with and without the MR‐MinMo. Group statistics were calculated by averaging these individual average values for each condition (MR‐MinMo and SP) on a per participant basis.

We investigated the relationship between average motion for translation and rotation and the improvement in NGS after motion correction as a proxy for reconstruction success. The average motion (translation and rotation) for each participant was plotted against improvement in NGS. We also estimated a linear regression (using MATLAB's *polyfit.m*) for the improvement in NGS post retrospective motion correction against the average motion for translation and rotation and plotted the line of best fit for the MR‐MinMo and standard padding groups.

### 2.9 T2* Map Analysis

T2* maps were calculated voxel‐by‐voxel for the multi‐echo linearly acquired data using our own scripts written in MATLAB. T2* fitting was done using the *robustfit.m* function and involved using the T1‐weighted signal from all the echoes in the GRE acquisition to obtain the $T2^{*}$ map [22]. This was further validated as a measure of motion related image degradation by Castella et al. [11] who found that the standard deviation of R2*—SD(R2*) values—within white matter (WM) closely related to aggregate measures of motion from a camera tracking system. We therefore followed a similar approach and calculated SD(R2*) within white matter, where larger values indicate greater motion degradation. White matter was segmented as described above using the same tissue classification approach on first echo images, with probability maps thresholded at 0.9 and processed using the same morphological closing (disk element radius 2), followed by voxel erosion (disk element 2) to exclude partial volume regions at tissue boundaries and minimize contributions from regions with strong B0 inhomogeneities. T2* maps were converted to R2*, outliers (values outside the range 5–200 s^−1^) were removed, and SD(R2*) was computed within each subject's white matter mask. Lower SD(R2*) values indicate improved precision attributable to reduced motion artifacts.

## 3 Results

### 3.1 MR‐MinMo Efficacy

**FIGURE 4:**
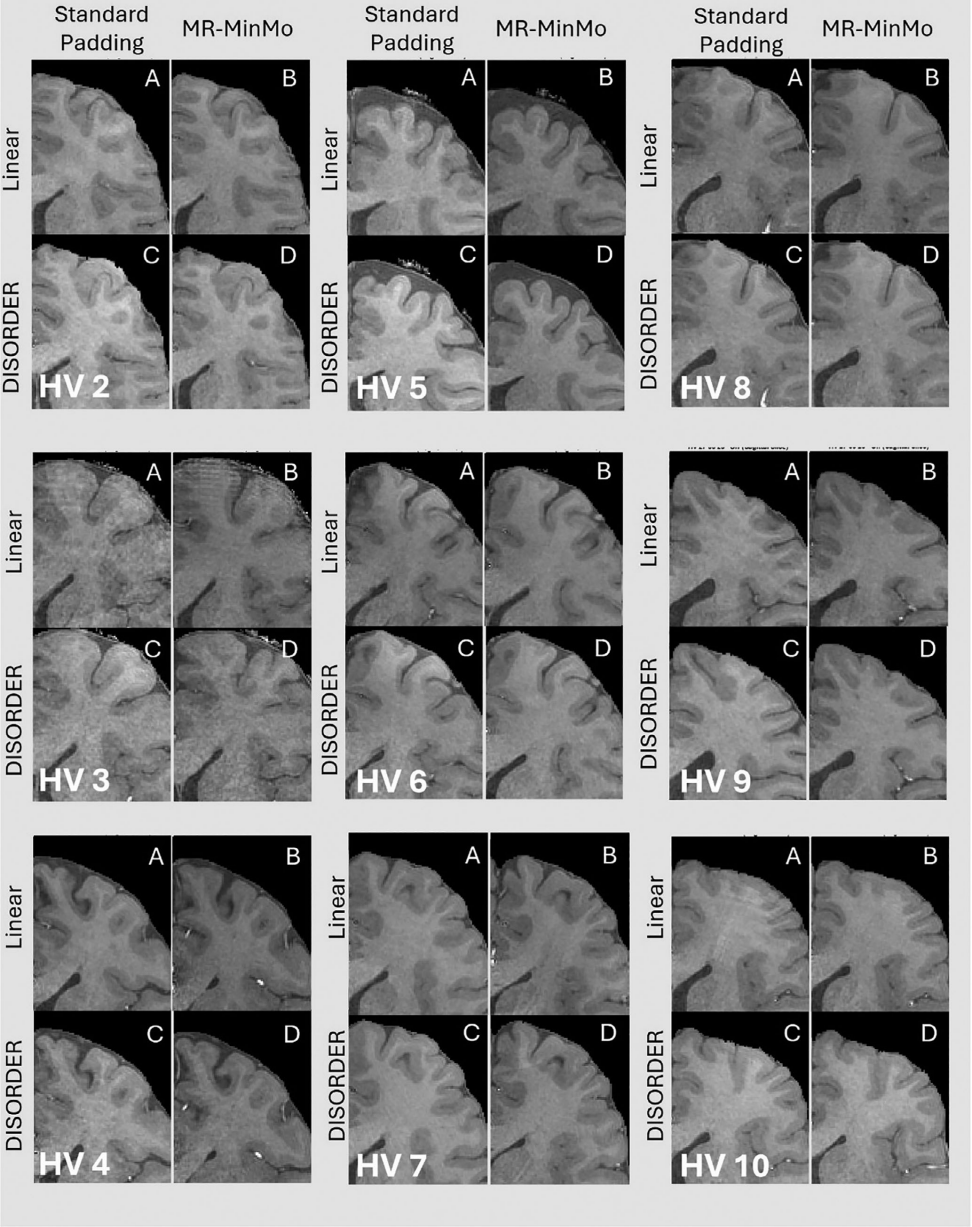
Representative images showing the standard padding (SP) and MR‐MinMo conditions for the linear sampling and retrospectively motion corrected DISORDER sampled scans for nine adults HVs (HV2‐HV10). Images shown are zoomed regions of transverse orientation slices to facilitate visualization of motion artifact levels. The images were selected visually to display the closest equivalent anatomical area without performing coregistration between SP and MR‐MinMo conditions that required subject repositioning.

**FIGURE 5:**
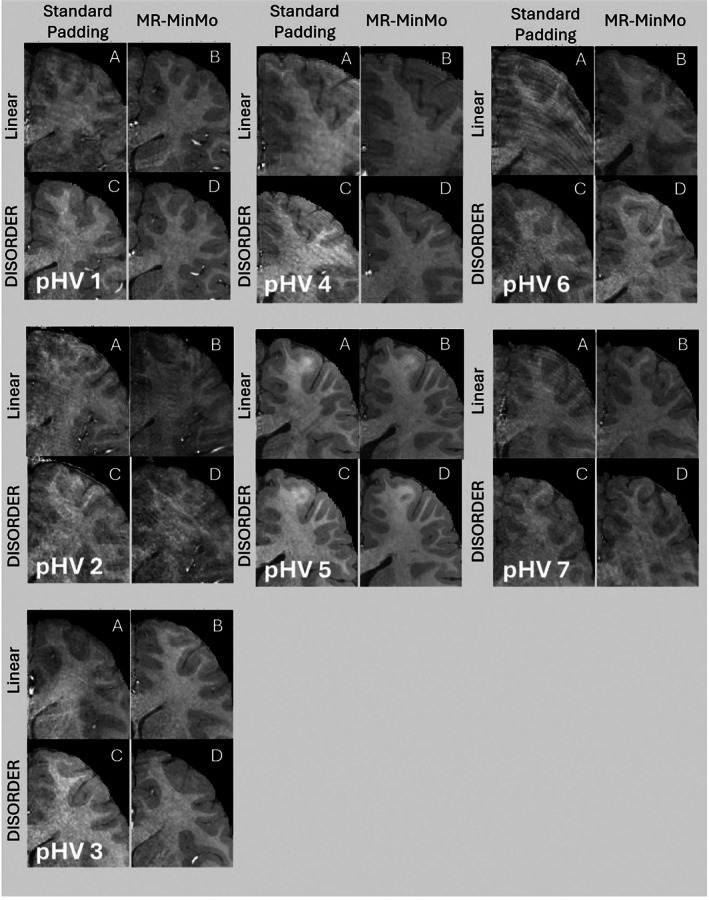
Representative images showing the standard padding (SP) and MR‐MinMo conditions for the linear sampling and retrospectively motion corrected DISORDER sampled scans for seven pediatric HVs (pHV1‐7). Images shown are zoomed regions of transverse orientation slices to facilitate visualization of motion artifact levels. The images were selected visually to display the closest equivalent anatomical area without performing coregistration between SP and MR‐MinMo conditions that required subject repositioning.

MR‐MinMo images were visually improved in nearly all subjects both for the long duration linearly sampled acquisition and for the very long duration DISORDER retrospective motion correction images. This can be seen in Figures 4 and 5 that show the bias field corrected first echo image of the nine adults and seven children, for the MR‐MinMo (B and D) and standard padding conditions (A and C). The images show the zoomed‐in regions near the frontal cortex for all participants where motion can be most clearly visualized. While motion artifacts were visually reduced for 14 out of 16 when the MR‐MinMo device was used, the improvement in image quality was more apparent in pediatric HVs. Since the position within the RF coil of the HV had changed between the MR‐MinMo and standard padded scans, slices with the closest visual appearance without any explicit co‐registration are shown to avoid interpolation effects. In two (out of seven) pediatric participants (pHV2, pHV7 in Figure 5) DISORDER motion‐corrected scans showed degraded image quality (Box “D”) compared to linear scans (Box “B”). Motion state estimates for each individual (see Table S1) are only available for the DISORDER scans; however, this showed that these participants had greater motion. pHV7 showed the largest average rotation with SP, and pHV2 showed the second largest average translation with SP among all participants.

### 3.2 Quantitative Image Quality Analyses

**FIGURE 6:**
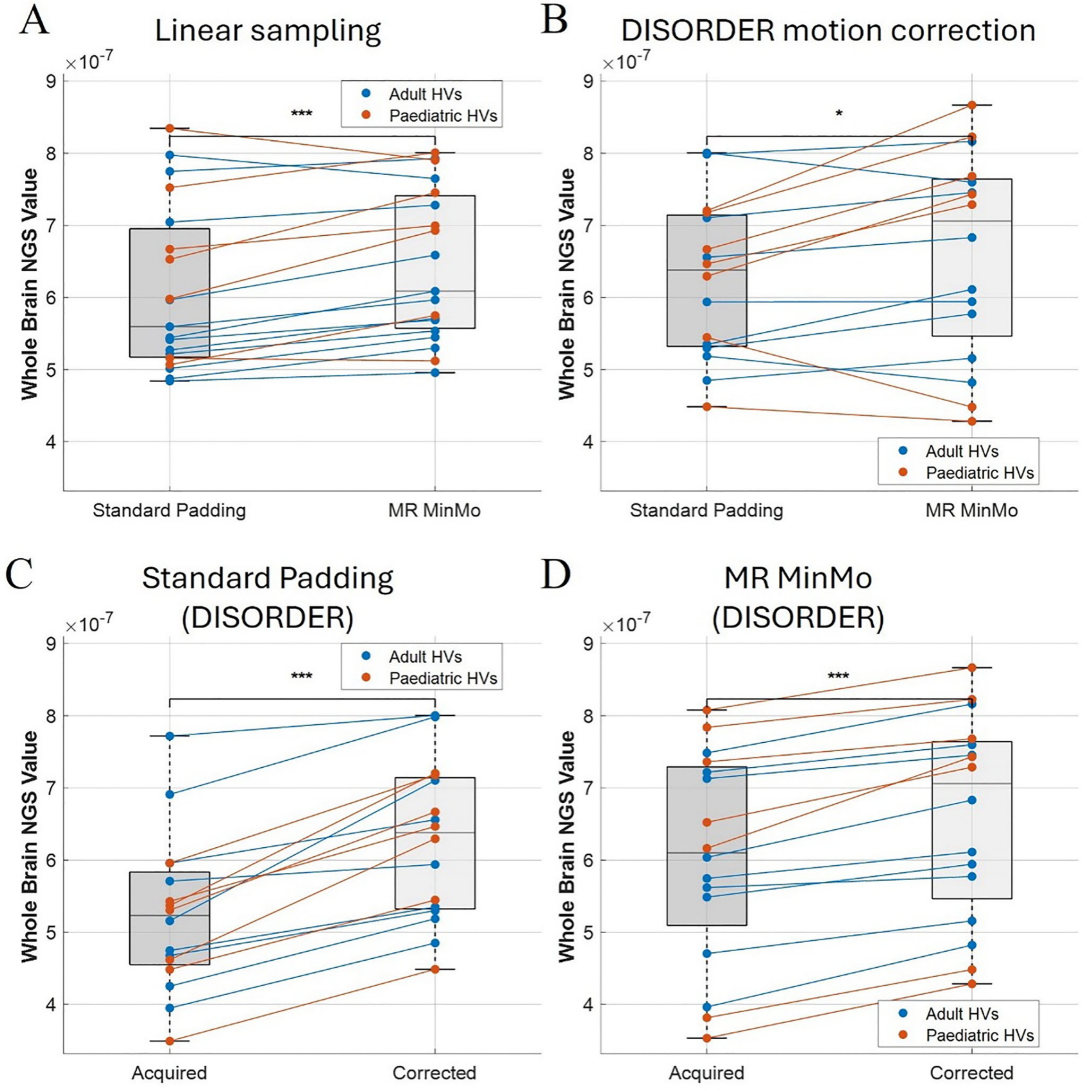
Boxplots of the whole‐brain NGS values for all participants, comparing the SP and MinMo conditions in (A) which shows the results for the linear sampling acquisition and (B) for the motion corrected DISORDER case; Also comparing the DISORDER acquired and DISORDER motion corrected cases (C) for the standard padding case and the (D) for the MR‐MinMo case. The connected lines represent the change in image quality between the different conditions for adult (blue) and pediatric HVs (red) separately, with an increase in NGS indicating improved image quality. The boxplots display the median NGS value, while the box boundaries represent the first and third quartile range. Asterisks denote statistical significance (*p < 0.05, ***p < 0.001).

To quantify the artifact levels in the ME‐GRE images shown above, the whole brain NGS values were calculated for all the HVs and the experimental conditions are shown graphically in Figure 6. Figure 6A,B shows the NGS values calculated from the images acquired with linear sampling and DISORDER motion correction, respectively. Each plot shows every individual's NGS values paired between standard pads and MR‐MinMo. It can be seen that the NGS values are increased, on average and for most individuals for both linear and DISORDER scans. Figure 6C shows the NGS values calculated from the images acquired with standard padding and the DISORDER trajectory prior to and post retrospective motion correction. This demonstrates that the reconstruction was effective in reducing motion in the DISORDER acquired images. Finally, in Figure 6D the NGS values were calculated prior to and post DISORDER‐based retrospective motion correction of images acquired with the MR‐MinMo. Comparing between Figure 6C,D it can be seen that the MR‐MinMo NGS values in Figure 6D are increased compared to the standard padding and that the combination of MR‐MinMo and correction provides an increased NGS value compared to the retrospective correction or the MR‐MinMo used individually. These differences were statistically significant; the two‐way repeated measures ANOVA with age as a binary factor showed that there was a significant main effect of MR‐MinMo on the NGS values (*F* = 34.393, *p* < 0.001). A significant interaction was found between MR‐MinMo and age group (*F* = 8.311, *p* = 0.012), and a significant three‐way interaction between MR‐MinMo, DISORDER and age group (*F* = 5.599, *p* = 0.033) indicating differential effects of the MR‐MinMo device across age groups and sampling types. Overall, this implies that DISORDER was more effective when the range of motion was reduced by the MR‐MinMo device. DISORDER motion correction efficacy was assessed by comparing NGS values before and after retrospective correction using paired *t*‐tests. As expected, retrospective motion correction significantly improved DISORDER scans across subject age groups and head stabilization conditions: adult HVs standard padding (*p* = 0.0016), adult HVs MR‐MinMo (*p* = 0.0002), pHVs standard padding (*p* < 0.0001), and pHVs MR‐MinMo (*p* = 0.0012).

### 3.3 Motion Quantification

As calculated from the motion states estimated by DISORDER, MR‐MinMo provided substantial motion reduction in both volunteer groups, with greater effects observed in pediatric participants (Table 1). Overall, MR‐MinMo reduced average translation motion by 43.1% (from 0.325 to 0.185 mm) and average rotation by 36.6% (from 0.190° to 0.121°). Pediatric HVs showed larger motion reductions (translation: 52.2% reduction from 0.498 to 0.238 mm; rotation: 54.4% reduction from 0.288° to 0.131°) compared to adult HVs (translation: 24.7% reduction from 0.191 to 0.143 mm; rotation: 1.8% reduction from 0.115° to 0.113°). These measurements indicate that pediatric participants moved substantially more than adults during the DISORDER sampled acquisition, but both groups benefited substantially from MR‐MinMo.

**TABLE 1:** Average motion for HVs for translation and rotation are presented for standard padding (SP) and MR‐MinMo (MR‐M) conditions, with percentage reduction (Red%) indicating the motion reduction achieved with the MR‐MinMo device.

	Average rotation (°)			Average translation (mm)		
	SP	MR‐M	Red%	SP	MR‐M	Red%
Adult	0.115	0.113	1.8	0.191	0.143	24.7
Pediatric	0.288	0.131	54.4	0.498	0.238	52.2
All	0.190	0.121	36.6	0.325	0.185	43.1

*Note*: Individual volunteer rotations and translations were averaged within each group (Adult, Pediatric) and across the combined group (All) for the group statistics.

Motion estimates were also compared between the first and second halves of the DISORDER acquisition (48 states each) to assess whether motion increased over time in the 20‐min long scan. No significant differences were found for either standard padding (average translation: 0.222 vs. 0.225 mm, *p* = 0.47; average rotation: 0.125° vs. 0.130°, *p* = 0.92) or MR‐MinMo (average translation: 0.136 vs. 0.120 mm, *p* = 0.30; average rotation: 0.089° vs. 0.077°, *p* = 0.61).

**FIGURE 7:**
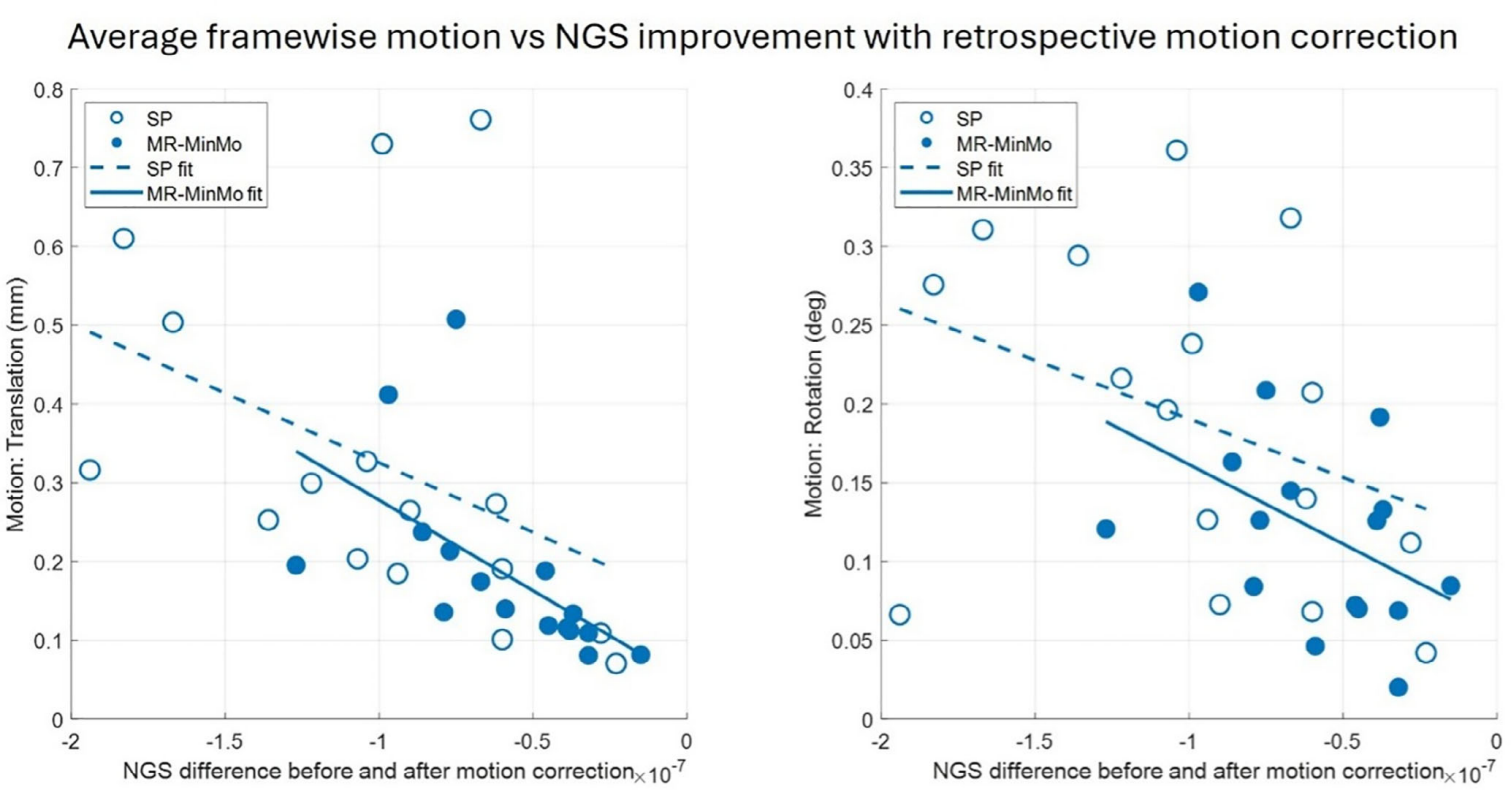
Plots showing the relationship between average temporal standard deviation in translation and rotation parameters and the NGS difference before and after motion correction for both the standard padding and MR‐MinMo condition. For each of the 16 participants with DISORDER sampled acquisitions the open circles represent Standard padding and the filled circles MR‐MinMo conditions, respectively. The dashed line shows the linear best fit for SP points and the solid line the best fit for the MR‐MinMo condition.

All participants showed an improvement in NGS following correction for both standard padding and MR‐MinMo cases (Figure 7). There was a linear association between NGS improvement after motion correction and degree of intrascan motion. There was a higher gradient between intrascan motion and NGS improvement for the MR‐MinMo condition consistent with the significant interaction found statistically. There was also a reduction in the mean absolute residuals: MR‐MinMo: 0.057 mm/0.047° and SP: 0.139 mm/0.074° (Table S2 for detailed statistics).

### 3.4 T2* Maps

**FIGURE 8:**
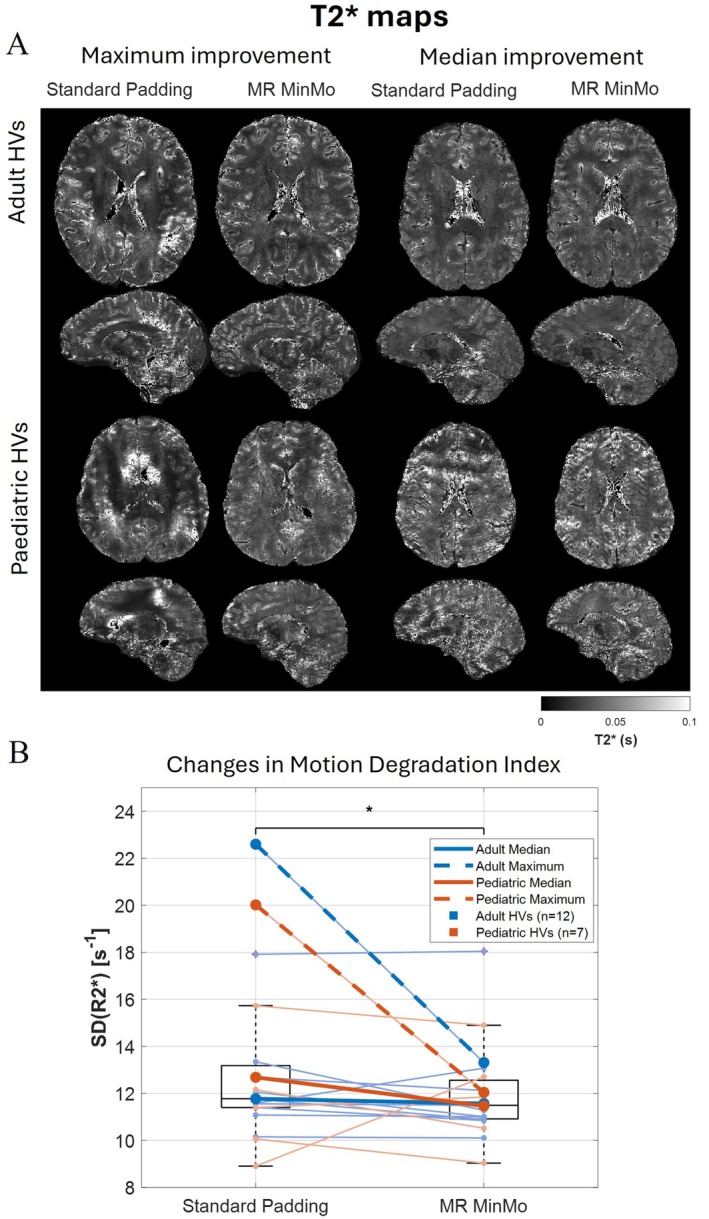
(A) Representative T2* maps (axial and sagittal views) shown for Standard Padding and MR‐MinMo cases obtained by fitting across the ME‐GRE echoes of the linearly sampled acquisition for the adult HV and pediatric HV with the maximum improvement and median improvement in SD(R2*) values. (B) Boxplot showing the motion degradation index SD(R2*) for standard padding and MR‐MinMo conditions across adult (blue) and pediatric (red) participants. Individual subject trajectories are shown with light connecting lines, while representative cases with median (solid lines) and maximum (dashed lines) improvement are highlighted. Statistical significance is indicated by asterisk (paired *t*‐test done for all HVs).

Motion precision was quantified using the motion degradation index SD(R2*) within white matter across all volunteers. MR‐MinMo demonstrated significantly improved T2* maps compared to standard padding (as shown in Figure 8A), with mean SD(R2*) values across the group of 12.0 s^−1^ for MR‐MinMo versus 13.1 s^−1^ for standard padding (*p* < 0.05, paired *t*‐test). The significantly lower standard deviation with MR‐MinMo (12.0 vs. 13.1 s^−1^) indicated more consistent results across all volunteers, demonstrating reduced motion‐related variability in R2* measurements.

Representative participants, as shown in Figure 8B, benefited from a range of improvement, with adult HVs showing 2% (median) to 41% (maximum) SD(R2*) reduction, while pediatric HVs demonstrated 10% (median) to 40% (maximum) reduction. The significant maximum improvements in both groups show the ability for MR‐MinMo to facilitate quantitative and qualitative motion suppression in select cases, while median improvements represent more general expected results across the cohort of volunteers.

## 4 Discussion

We demonstrated that the MR‐MinMo device was highly effective in reducing movement in the context of long‐duration 7 T scans. There was clear improvement in image quality both visually and quantitatively for scans of either ∼10 min or ∼20 min duration. Measures of motion estimated from retrospective correction showed significant reductions in the average amount of temporal motion. The MR‐MinMo was effective across participants in both compliant adult volunteers and younger pediatric volunteers. The younger group, who were also MRI naïve, tended to move more than older volunteers and therefore the MR‐MinMo had the largest effect on image quality in this group. Quantitative R2* maps from the linear sampling protocol demonstrated a significant reduction in motion artifacts and increased precision, consistent with improvements in ME‐GRE images. The 0.6 mm isotropic resolution ME‐GRE scans are typical of those used in quantitative multiparametric mapping studies [23, 24] at 7 T, demonstrating MR‐MinMo effectiveness in this context.

DISORDER sampling scans with 20‐min duration are deliberately motion sensitized to allow for motion estimation and subsequent correction. Retrospective correction significantly improved image quality, and we found an interaction between MR‐MinMo use and retrospective correction suggesting synergistic effects. We found that DISORDER scans (pHV2, pHV7 in Figure 5) that showed degraded image quality had large movement, which is consistent with the idea that reducing motion may maintain data within a correctable regime (Table S1). We also visualized the effect motion correction on NGS values compared to average framewise motion (Figure 7), demonstrating that the MR‐MinMo increased the effectiveness (gradient between Framewise motion and NGS improvement) and consistency (reduced residuals) of the retrospective correction. This is broadly consistent with our hypothesis that the MR‐MinMo kept the motion within a correctable regime. Alternative motion correction methods using navigators [25, 26, 27] or tracking systems [1, 28, 29, 30] reliant on additional hardware/software may similarly also benefit from reduced head pose variation.

Accurate coil sensitivity estimation is crucial for robust parallel imaging reconstruction [31] but Participant motion affects receiver sensitivity profiles, particularly near small, dense receiver coil arrays, introducing potential reconstruction errors [6]. Movement can also affect B1+ field variability and while this is typically considered a minor contributor to reconstruction errors, minimizing motion‐related variance at the source can help reduce any residual variance. Suppressing motion with MR‐MinMo helps reduce this error source, explaining the significant interaction between MR‐MinMo and retrospective motion correction. However, we did not directly assess these factors.

Previous immobilization attempts include devices such as vacuum cushions that may be effective in various contexts [32, 33, 34, 35, 36, 37, 38] but that are not routinely used despite availability for many years. Their efficacy for high resolution 7 T imaging has not been demonstrated to our knowledge although anecdotally, vacuum cushions have been used in some centers and may potentially provide motion reduction benefits, and we cannot exclude the possibility that vacuum cushions might achieve similar motion reduction to MR‐MinMo without a direct comparison.

Bite bars [39] and individualized head molds or thermoplastic masks [40] have also been used to reduce head movement. Custom individualized foam inserts show mixed results with reports of both effectiveness and limited efficacy [41, 42]. Bite bars and thermoplastic masks require custom manufacturing limiting deployment and integration into clinical workflows with additional issues of increased discomfort, feelings of claustrophobia, and a sense of constriction, potentially exacerbating movement especially in pediatric use. This is problematic for long scans such as high‐resolution whole brain structural MRI at 7 T. We demonstrated encouraging results with clear improvements in adults and children using a device customizable in situ across a wide age range. In this context, we have shown encouraging results with clear improvements in image quality in adults and children with a device customizable in situ to the individual across a wide age range. Due to space restrictions within the RF coil, subjects with larger head sizes may not be positioned comfortably with or without MR‐MinMo.

Other motion mitigation strategies involve real‐time tracking of head position using MRI acquisition adaptations (navigators), external devices such as optical cameras [43, 44], acoustic or ultrasound sensors [45, 46, 47], inertial/mechanical devices [48, 49], or RF‐sensor‐based hardware [50] showing improvements in motion‐related artifacts. However, practical limitations have restricted routine use [51]. Should these be overcome, these strategies can be employed, and are complementary to improved head stabilization.

### 4.1 Limitations

Most adult HVs that were scanned were accustomed to 7 T scanning; however, all pediatric HVs were scanned for the first time. We did not formally record subject experience of comfort in this study because data acquisition performed using institutional ethics approval for general MRI methodology development. This broad ethics framework, applicable across diverse technical studies, precluded sub‐study specific surveys. However, anecdotal feedback from participants mostly reported increased comfort with the MR‐MinMo, consistent with reduced movement in very long scans estimated by DISORDER framework because reduced movement is only likely if subjects were comfortable. Future systematic feedback with appropriate study‐specific ethics approval will be required to confirm this.

In all adult HVs except one, the MR‐MinMo latch successfully closed for scanning in closed configuration. For one adult HV with larger head circumference, the Halo was removed and scanning proceeded in the open configuration. This volunteer (HV3) showed increased motion artifacts in the 20‐min scan with MR‐MinMo compared to standard padding; however, the DISORDER framework still increased image quality. Adult participants were also given the option to watch TV; however, some reported difficulty viewing the full screen extent while the MR‐MinMo was in use due to standard mirror size and positioning limitations, which could be overcome with a custom mirror.

In Figure 8 that corresponds to the linearly sampled acquisition results, one adult HV showed degradation in T2* maps (SD(R2*)–18 s^−1^) in both standard padding and MR‐MinMo conditions with minimal improvement from the device. We note that this was a large participant (male, age 26, height 6′1″) where the MR‐MinMo is likely to be less effective owing to space constraints that can reduce comfort while it was also used second in this individual relative to SP. Additionally, it is possible the limited FOV reference scan used for coil sensitivity estimation may have introduced artifacts for this subject with a larger head size that propagated through coil sensitivity maps, degrading reconstruction quality for all subsequent acquisitions regardless of stabilization. One pediatric HV showed T2* map quality degradation with the MR‐MinMo. For this participant, the scan with MR‐MinMo was acquired last in the session, and cumulative fatigue combined with pediatric motion propensity likely contributed to the elevated degradation hence why scan order was randomized.

For pediatric HVs, pHV2 and pHV7 showed degraded motion quality with the motion‐corrected longer scan compared to the shorter standard linear scan; these participants' motion estimates suggest that large motion events occurred during the long duration DISORDER scan. Although we do not have motion estimates from the linear scan presumably, they had less motion. This could suggest that imaging faster is preferable to correction; the specific DISORDER motion correction strategy used in this study used a conservative acceleration factor (overall *R*≈2) previously found necessary for robust motion correction [13]. However, more generally, alternatives or augmentations (e.g. [52]) to DISORDER may be able to achieve similar scan durations using higher acceleration with retrospective motion correction. The aim of this paper was not to judge the utility of the DISORDER vs. linear acquisition but rather judge the utility of the MR‐MinMo device in high resolution scans with and without motion correction with the additional advantage of the latter providing motion state estimation. For visual comparison, we used native images without coregistration between conditions. While anatomy is not identical, this maintains fidelity regarding PE directions differentially sensitive to motion artifacts while avoiding potential interpolation artifacts such as partial volume effects or overshooting/ringing artifacts [53].

Some residual artifacts potentially pertaining to Gibb's ringing may remain in some HVs but were consistent across conditions and could be removed by *k*‐space filtering techniques. We avoided filtering of any kind for clearest visualization and measurement of any differences between standard and MR‐MinMo stabilization.

We evaluated intrascan motion, but many quantitative protocols (e.g., MPM [23, 54]) consist of acquiring several images with different parameters (e.g., flip angle). Although not evaluated here, reductions in interscan movement might improve quantitative mapping by reducing position‐dependent transmit and receive field effects [55]—this should be explored in future studies.

The pediatric cohort in this study was limited to seven volunteers. While we observed statistically significant motion reduction effects and improvement in image quality with MR‐MinMo in the pediatric group indicating a large effect size (translation reduction: 52.2%, rotation reduction: 54.4%), the small sample size and single center results from the study should be taken as providing a preliminary proof‐of‐concept for pediatric application of the MR‐MinMo device at 7 T. Future larger‐scale studies with bigger cohorts at different centers are needed to confirm these findings.

The MR‐MinMo use was randomized across all HVs; 9 of 19 HVs were scanned with the MR‐MinMo first, 10 with MR‐MinMo second, ensuring that any increase in motion prevalence with time in the scanner [20] should not have affected results while minimizing the effect of motion profile changes in a given sampling's scans with and without MR‐MinMo.

No direct comparison was made to other head stabilization methods, such as vacuum cushions, so relative efficacy cannot be unambiguously determined. Future studies may directly compare the MR‐MinMo with vacuum cushions in different imaging centers and subject larger populations to generalize and confirm optimal motion mitigation strategies in different contexts. None‐the‐less comparison to current practice showed clear advantages in image quality from improved head stabilization with and without retrospective correction.

## 5 Conclusion

The MR‐MinMo was found to be an effective solution for reducing head motion and increasing image quality in high‐resolution 7 T scans in adults and particularly children. The device is compatible with motion correction methods and was shown to improve the performance of the DISORDER retrospective motion correction method.

## Funding

This work was supported by EPSRC smart medical imaging CDT studentship (Dr Jyoti Mangal, EP/S022104/1), Wellcome/EPSRC Centre for Medical Engineering [WT203148/Z/16/Z], and National Institute for Health Research (NIHR) Biomedical Research Centre based at Guy's and St Thomas' NHS Foundation Trust and King's College London.

## Supporting information


**Table S1:** Average of the temporal standard deviation of the motion states across HVs for translation and rotation are presented for standard padding (SP) and MR‐MinMo (MR‐M) conditions.
**Table S2:** Linear regression statistics presented for the motion versus NGS difference plot given in Figure 7. Slope indicates the change in motion (mm or degrees) per unit change in NGS change. *R* ^2^ indicates the proportion of variance in motion explained by correction efficacy; mean absolute residual indicates average deviation from the fitted line. Mean_Abs_Residual, Mean of absolute residual values; MRM, MR‐MinMo; R_squared, coefficient of determination; SP, standard padding.
**Figure S1:** Photos of MR‐MinMo device with and without subject inside the Nova 8‐channel PTx coil. Top row shows the MR‐MinMo device in both open and closed configuration. Bottom row shows the subject loaded in the device with the coil in the loaded and unloaded positions.
**Figure S2:** Overlay showing relative patient position with and without the MR‐MinMo device in a representative adult and a representative pediatric subject. The magenta and green color coding show the images acquired without and with the device, respectively, with the scale showing the movement of subject between the two is within ∼1 cm.

## Data Availability

Representative T2* quantitative maps and analysis code are available on URL upon request. The MR‐MinMo device design specifications are available from the corresponding author upon reasonable request. Source code for the DISORDER reconstruction and motion analysis is available at URL on request. Raw imaging data are available subject to ethical approval and data sharing agreements.
