# Supplementary material for: Reducing Motion Artifact in High Resolution 7 T MRI Using the Magnetic Resonance Minimal Motion (“MR‐MinMo”) Head Stabilization Device
Source: Magn Reson Med. 2026 Jun 2;96(3):1486–501. doi: 10.1002/mrm.70424 (PMC13327473; doi:10.1002/mrm.70424)
Supplement: Supplementary file 1 — Table S1: Average of the temporal standard deviation of the motion states across HVs for translation and rotation are presented for standard padding (SP) and MR‐MinMo (MR‐M) conditions. Table S2: Linear regression statistics presented for the motion versus NGS difference plot given in Figure 7. Slope indicates the change in motion (mm or degrees) per unit change in NGS change. R 2 indicates the proportion of variance in motion explained by correction efficacy; mean absolute residual indicates average deviation from the fitted line. Mean_Abs_Residual, Mean of absolute residual values; MRM, MR‐MinMo; R_squared, coefficient of determination; SP, standard padding. Figure S1: Photos of MR‐MinMo device with and without subject inside the Nova 8‐channel PTx coil. Top row shows the MR‐MinMo device in both open and closed configuration. Bottom row shows the subject loaded in the device with the coil in the loaded and unloaded positions. Figure S2: Overlay showing relative patient position with and without the MR‐MinMo device in a representative adult and a representative pediatric subject. The magenta and green color coding show the images acquired without and with the device, respectively, with the scale showing the movement of subject between the two is within ∼1 cm. [file MRM-96-1486-s001.docx]

Supplementary Information

|  | **Average Rotation (°)** | | **Average Translation (mm)** | |
| --- | --- | --- | --- | --- |
|  | **SP** | **MR-M** | **SP** | **MR-M** |
| HV_2 | 0.112 | 0.192 | 0.109 | 0.112 |
| HV_3 | 0.127 | 0.163 | 0.185 | 0.237 |
| HV_4 | 0.207 | 0.133 | 0.191 | 0.134 |
| HV_5 | 0.196 | 0.145 | 0.203 | 0.175 |
| HV_6 | 0.073 | 0.072 | 0.265 | 0.188 |
| HV_7 | 0.068 | 0.084 | 0.101 | 0.136 |
| HV_8 | 0.042 | 0.070 | 0.071 | 0.119 |
| HV_9 | 0.140 | 0.085 | 0.273 | 0.082 |
| HV_10 | 0.066 | 0.069 | 0.316 | 0.109 |
| pHV_1 | 0.311 | 0.121 | 0.504 | 0.195 |
| pHV_2 | 0.238 | 0.209 | 0.730 | 0.508 |
| pHV_3 | 0.276 | 0.046 | 0.610 | 0.140 |
| pHV_4 | 0.216 | 0.126 | 0.300 | 0.116 |
| pHV_5 | 0.294 | 0.020 | 0.253 | 0.081 |
| pHV_6 | 0.318 | 0.271 | 0.761 | 0.412 |
| pHV_7 | 0.361 | 0.126 | 0.327 | 0.213 |

Table S1 Average of the temporal standard deviation of the motion states across HVs for translation and rotation are presented for standard padding (SP) and MR-MinMo (MR-M) conditions

| **Statistic** | **Rotation (deg)** | **Translation (mm)** |
| --- | --- | --- |
| SP_Slope | -743111.8332 | -1764750.673 |
| SP_Intercept | 0.116245297 | 0.148943726 |
| SP_R_squared | 0.134489286 | 0.175206824 |
| SP_Mean_Abs_Residual | 0.074444125 | 0.139286032 |
| MRM_Slope | -1006990.09 | -2297829.51 |
| MRM_Intercept | 0.060878965 | 0.048176543 |
| MRM_R_squared | 0.205139352 | 0.327722474 |
| MRM_Mean_Abs_Residual | 0.047357103 | 0.057827294 |

Table S2 Linear regression statistics presented for the motion versus NGS difference plot given in Figure 7. Slope indicates the change in motion (mm or degrees) per unit change in NGS change. R² indicates the proportion of variance in motion explained by correction efficacy; mean absolute residual indicates average deviation from the fitted line. SP = Standard Padding; MRM = MR-MinMo; R_squared = coefficient of determination; Mean_Abs_Residual = Mean of absolute residual values


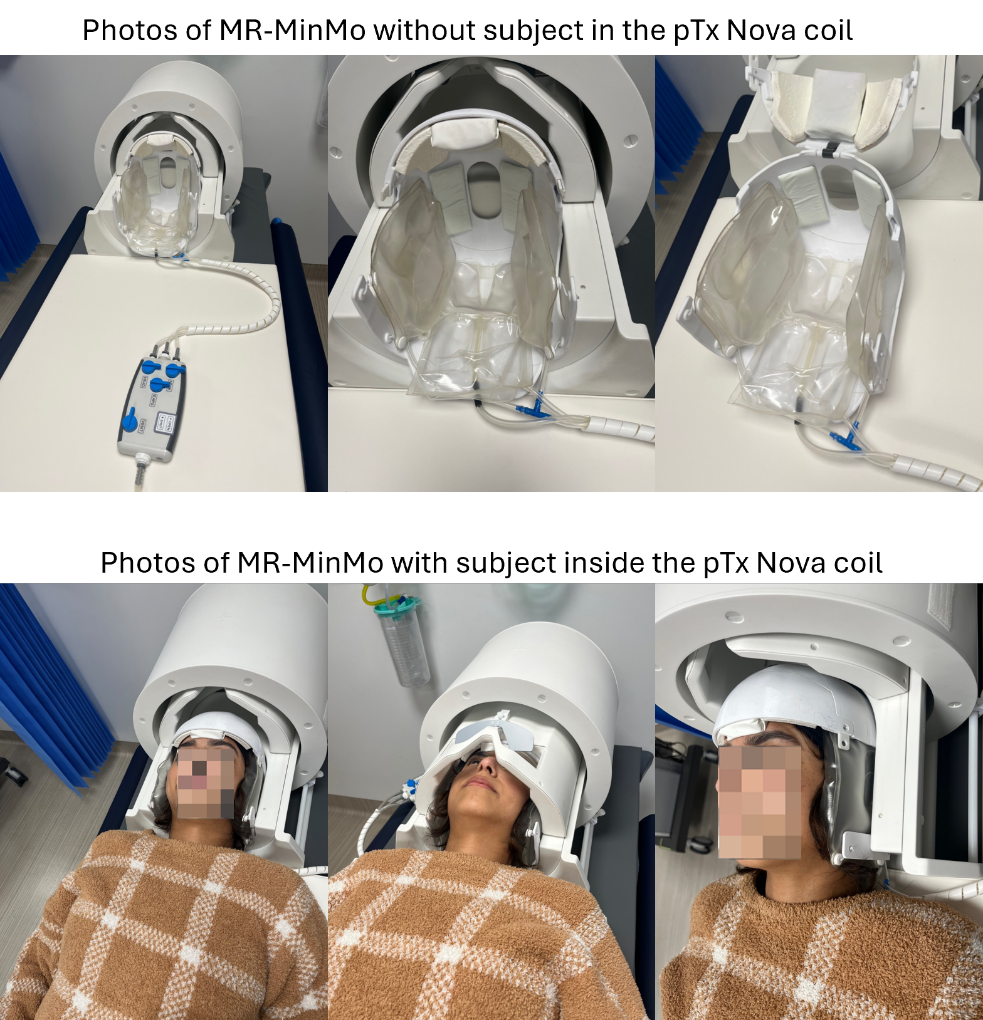


Figure S1 Photos of the MR-MinMo device with and without subject inside the Nova 8-channel PTx coil. Top row shows the MR-MinMo device in both open and closed configuration. Bottom row shows the subject loaded in the device with the coil in the loaded and unloaded positions.


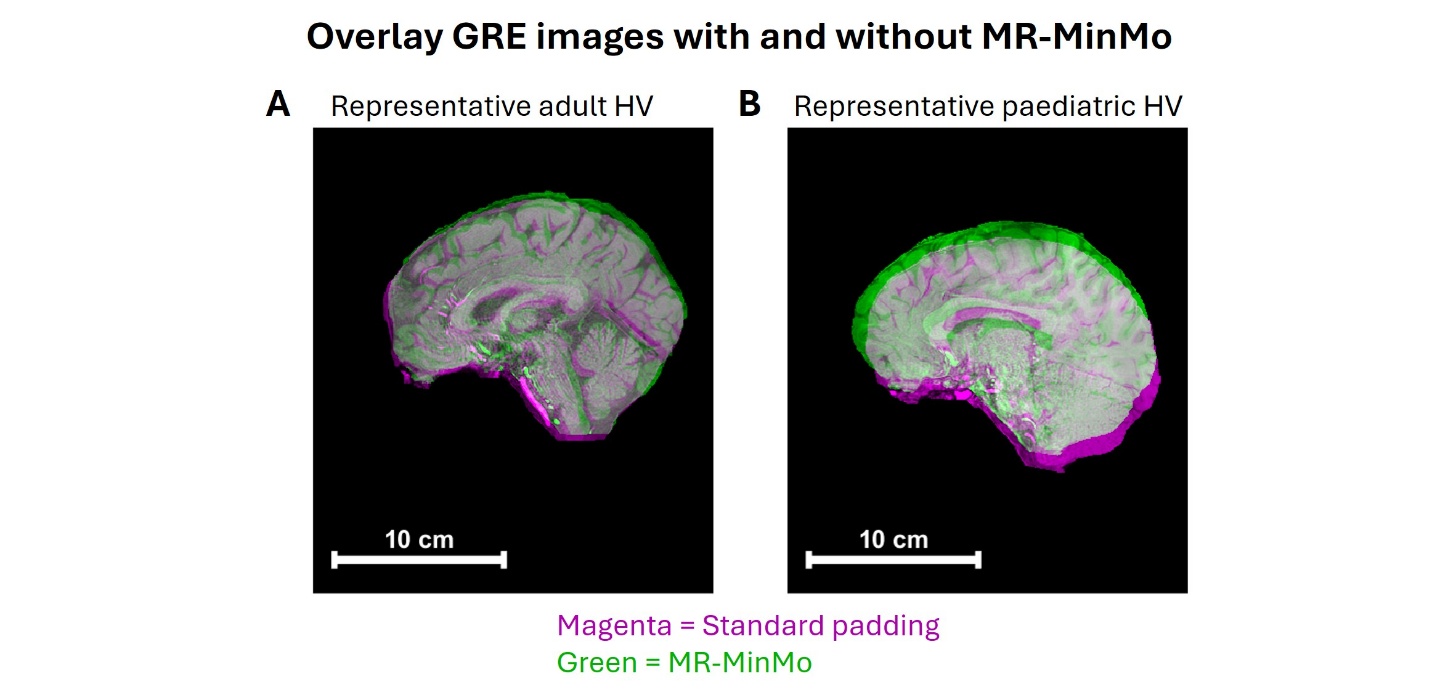


Figure S2 Overlay showing relative patient position with and without the MR-MinMo device in a representative adult and a representative paediatric subject. The magenta and green colour coding show the images acquired without and with the device respectively, with the scale showing the movement of subject between the two is within ~1cm.
